# Supplementary material for: Structural insights into small-molecule agonist recognition and activation of complement receptor C3aR
Source: EMBO J. 2025 Apr 7;44(10):2803–26. doi: 10.1038/s44318-025-00429-w (PMC12084609; doi:10.1038/s44318-025-00429-w)
Supplement: Supplementary file 1 — Appendix [file 44318_2025_429_MOESM1_ESM.pdf]

Appendix

Structural insights into small-molecule agonist recognition and activation of complement receptor C3aR

Jinuk Kim[1,†‡], Saebom Ko[1†], Chulwon Choi[1], Jungnam Bae[1], Hyeonsung Byeon[2], Chaok Seok[2], Hee-Jung Choi[1*]

[1]*Department of Biological Sciences, Seoul National University, Seoul 08826, Republic of Korea*
[2]*Department of Chemistry, Seoul National University, Seoul 08826, Republic of Korea*

† These authors contributed equally to this work.

‡ Present address: Division of Biological Science and Technology, Yonsei University, Wonju, 26493, Republic of Korea

* Correspondence:
Hee-Jung Choi, Ph.D.
E-mail: choihj@snu.ac.kr

Table of Contents:

Appendix Figure S1 Page 2

Appendix Figure S2 Page 3

Appendix Figure S3 Page 4

Appendix Figure S4 Page 5

Appendix Figure S5 Page 7

Appendix Figure S6 Page 8

Appendix Figure S7 Page 9

Appendix Table S1 Page 10

Appendix Table S2 Page 12

References Page 13

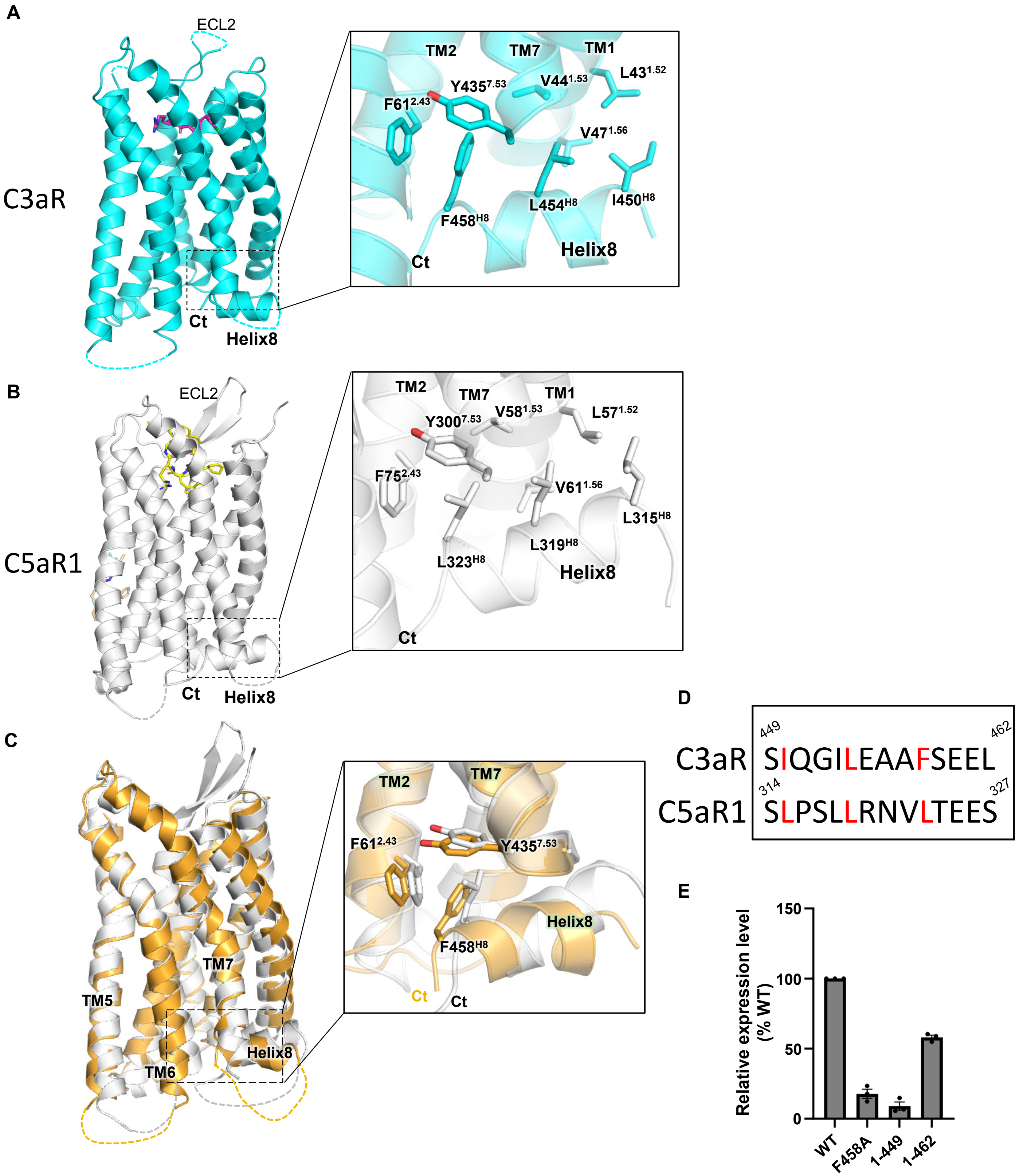

Appendix Figure S1 Inward face of the helix 8 of C3aR and C5aR1. A-B. Structures of JR14a-bound C3aR (cyan) and PMX53-bound C5aR1 (white; PDB: 6C1R) are shown. Hydrophobic interaction networks involving helix 8 are highlighted in the zoomed-in view on the right. Interacting residues are displayed as sticks. **C.** Structural alignment of apo-state C3aR (bright orange) and inactive, PMX53-bound C5aR1 (white) is shown, with a zoomed-in view on the right highlighting the hydrophobic interactions surrounding helix 8. **D.** Sequence alignment of helix 8 from C3aR and C5aR1. **E.** Quantification of surface expression levels of C3aR mutants, measured by surface ELISA. Bars represent the mean, and error bars indicate the standard error of the mean (S.E.M.) from three independent experiments.

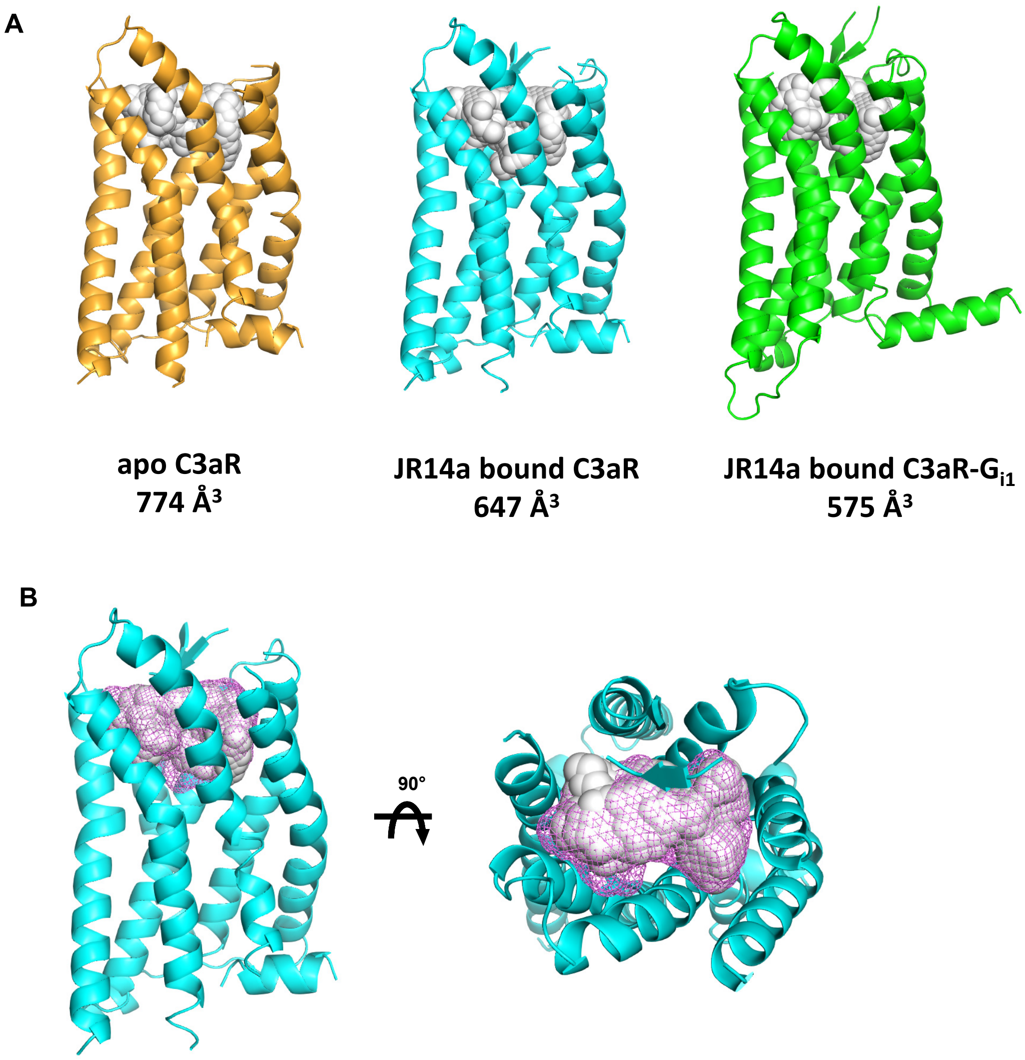

Appendix Figure S2. Orthosteric binding pocket volume of three states of C3aR. A. The orthosteric binding pocket volumes of C3aR in different conformational states, apo (bright orange), intermediate (cyan), and active (green), were calculated using POVME 2.0 (Durrant et al, 2011; Durrant et al, 2014) and are represented as white spheres. Calculated volumes for each state are provided below. **B.** Comparison of binding pocket volumes between the intermediate and active states. For visualization, the active-state volume is shown as white spheres overlaid on the intermediate-state structure, while the intermediate-state volume is represented as a magenta mesh.

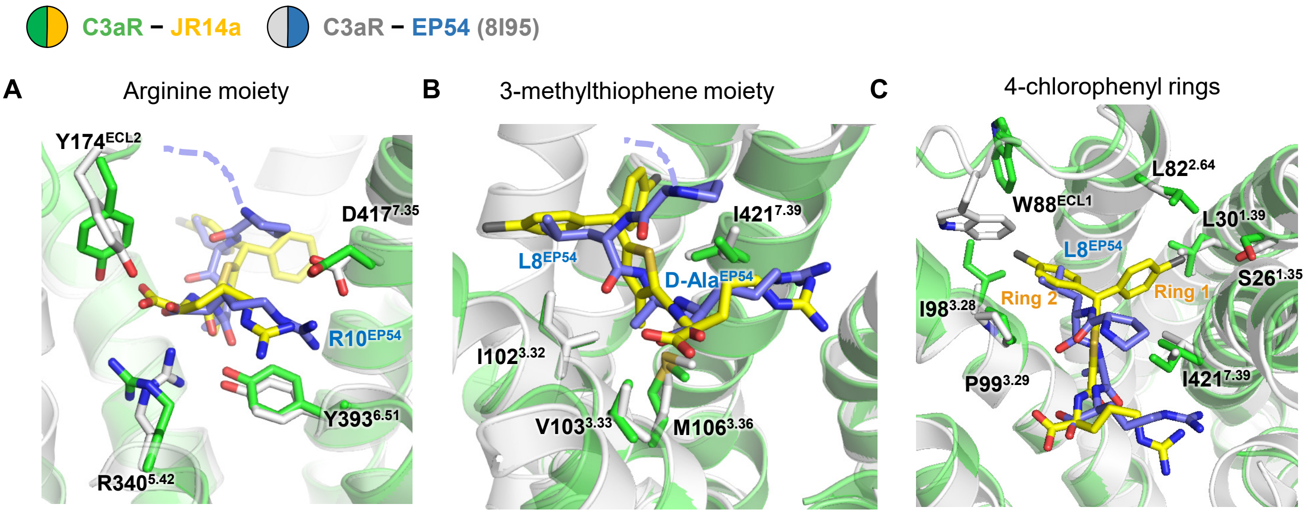

Appendix Figure S3. Comparison of binding modes of JR14a and EP54. A-C. Structural superposition of JR14a-bound active state C3aR with EP54-bound active state C3aR (PDB: 8I95). Residues interacting with each moiety of JR14a; **A.** C-terminal arginine, **B.** 3-methylthiophene, and **C.** 4-chlorophenyl rings are shown, respectively. For visual clarity, part of EP54 is replaced with dashed line or omitted.

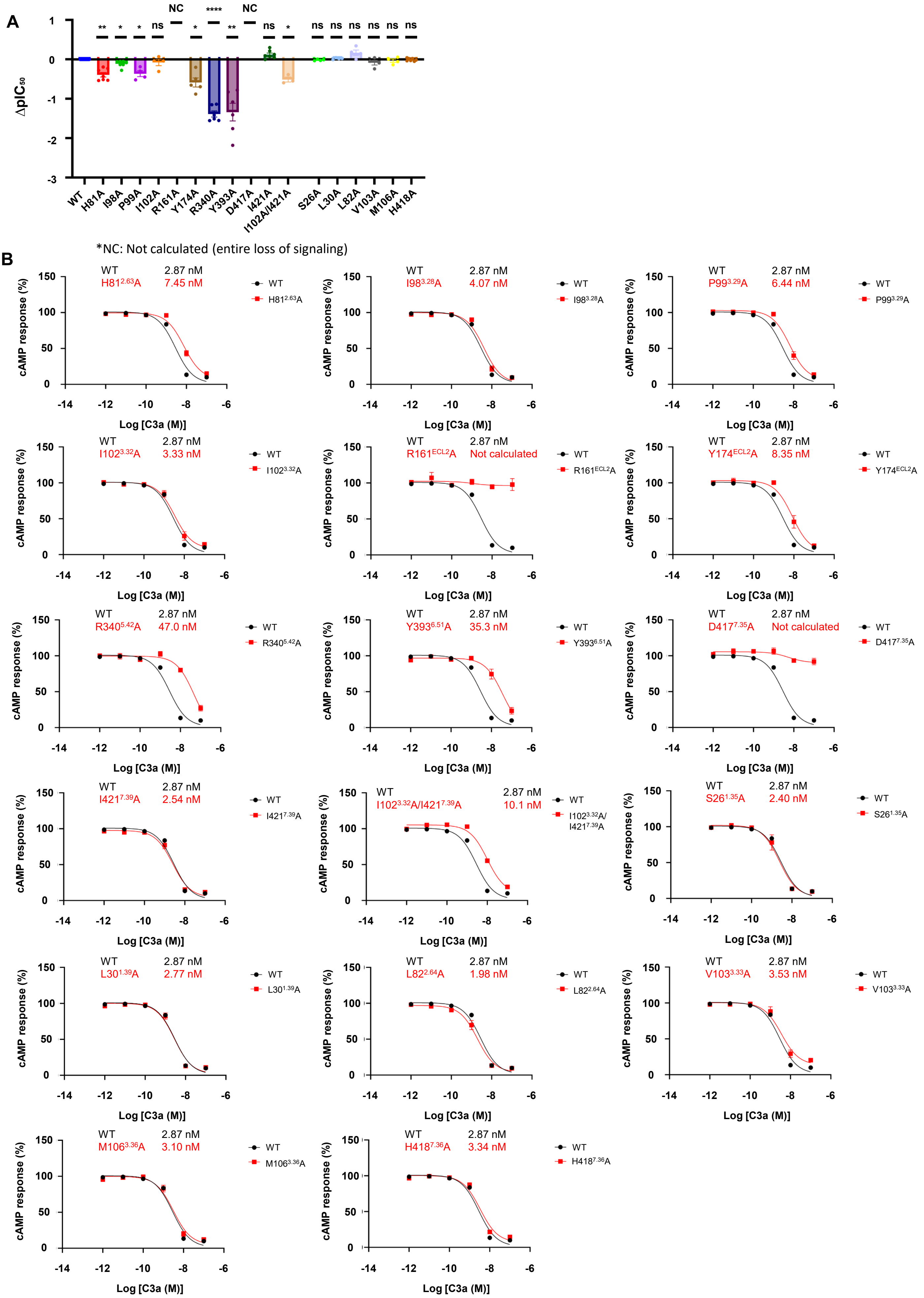
A
ΔpIC50
1
0
-1
-2
-3
WT
H81A
I98A
P99A
I102A
R161A
Y174A
R340A
Y393A
D417A
I421A
I102A/I421A
S26A
L30A
L82A
V103A
M106A
H418A
**
*
*
ns
NC
*
****
**
NC
ns
*
ns
ns
ns
ns
ns
ns
*NC: Not calculated (entire loss of signaling)
B
cAMP response (%)
Log [C3a (M)]
WT 2.87 nM
H81$^{2.63}$A 7.45 nM
I98$^{3.28}$A 4.07 nM
P99$^{3.29}$A 6.44 nM
I102$^{3.32}$A 3.33 nM
R161^{ECL2}A Not calculated
Y174^{ECL2}A 8.35 nM
R340$^{5.42}$A 47.0 nM
Y393$^{6.51}$A 35.3 nM
D417$^{7.35}$A Not calculated
I421$^{7.39}$A 2.54 nM
I102$^{3.32}$A/I421$^{7.39}$A 10.1 nM
S26$^{1.35}$A 2.40 nM
L30$^{1.39}$A 2.77 nM
L82$^{2.64}$A 1.98 nM
V103$^{3.33}$A 3.53 nM
M106$^{3.36}$A 3.10 nM
H418$^{7.36}$A 3.34 nM

Appendix Figure S4. Functional analysis of C3aR mutants in response to C3a via cAMP signaling. A. For each C3aR mutant, ΔpIC_{50} values were calculated relative to WT C3aR. 'NC' indicates that the value was not calculated. Bars and error bars indicate the means and the standard errors of the mean (S.E.M.) from 3-7 independent experiments, respectively. Statistical significance was assessed using one-way ANOVA followed by Dunnett's multiple comparisons test versus WT. ns $P>0.05$, * $P \leq 0.05$, ** $P \leq 0.01$, *** $P \leq 0.001$, **** $P \leq 0.0001$.

B. For individual plots, relative cAMP response (%) and IC_{50} values were calculated and compared to those of WT using GraphPad Prism 10.1.2. Data points and error bars indicate the means and the standard errors of the mean (S.E.M.) from 3-7 independent experiments, respectively.

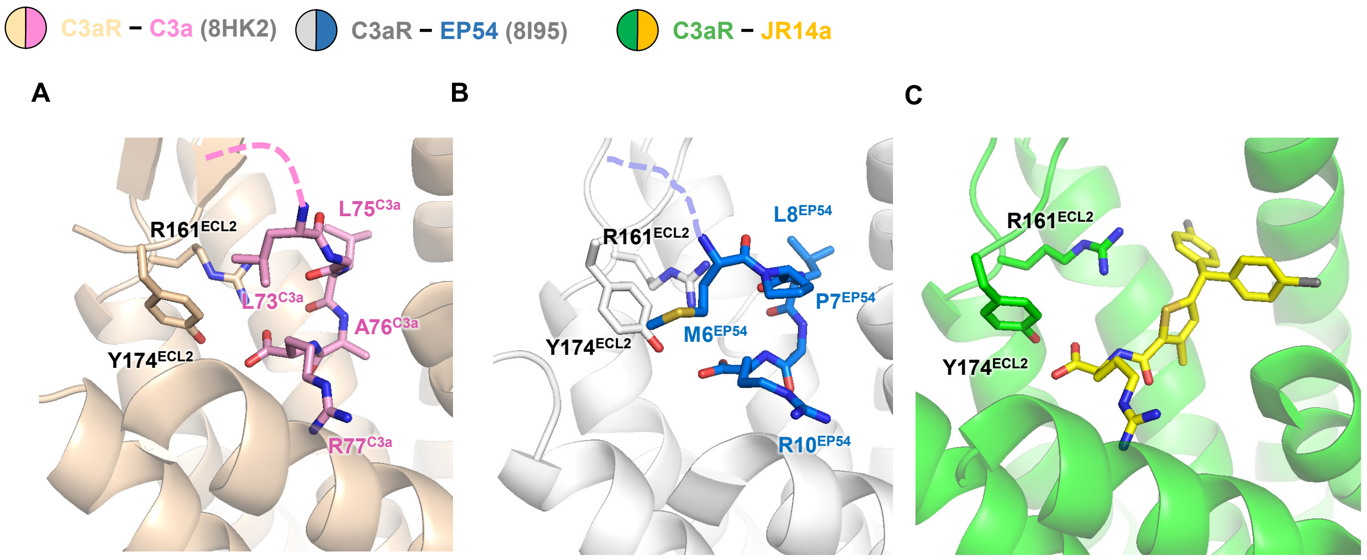

Appendix Figure S5. Comparison of Y174^{ECL2} and R161^{ECL2} interactions with C3a, EP54, and JR14a. A-C Structural comparison of JR14a-bound active state C3aR with C3a-bound (PDB: 8HK2) and EP54-bound (PDB: 8I95) C3aR. Residues interacting with Y174^{ECL2} and R161^{ECL2} are shown for each ligand. **A.** C3a, **B.** EP54, and **C.** JR14a. For visual clarity, portions of C3aR, C3a and EP54 are represented with dashed lines or omitted.

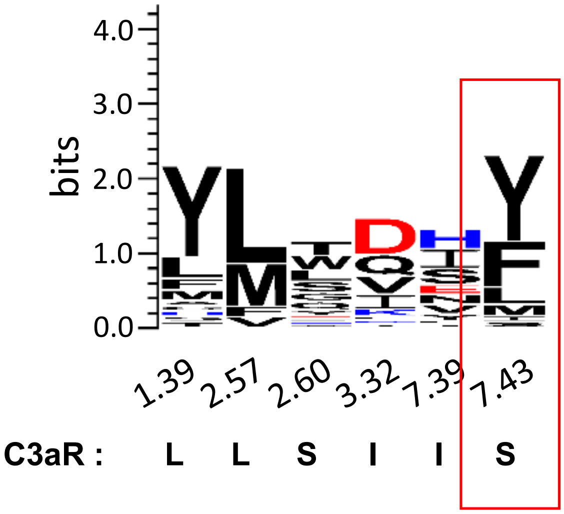

BW number	1.39	2.57	2.60	3.32	7.39	7.43
**C3aR**	**L**	**L**	**S**	**I**	**I**	**S**
C5aR1	F	L	L	I	V	Y
C5aR2	Y	L	L	I	V	L
FPR1	F	L	F	V	S	F
FPR2	L	L	L	V	S	F
FPR3	H	L	R	I	S	F
AT1R	Y	L	W	V	I	Y
AT2R	Y	L	W	L	I	F
CXCR1	Y	L	W	K	E	F
CXCR2	Y	L	W	K	E	I
CXCR4	Y	L	W	Y	E	F
δOR	Y	L	Q	D	I	Y
μOR	Y	L	Q	D	I	Y
κOR	Y	M	Q	D	I	Y
SST2R	Y	L	L	D	V	Y
NPFF1R	Y	M	T	Q	H	F
NPFF2R	Y	M	T	Q	H	F
OX1R	Y	L	S	Q	H	Y
OX2R	Y	L	T	Q	H	Y
NPY1R	Y	L	T	Q	H	M
NPY2R	Y	L	T	Q	H	M
5-HT4R	L	M	G	D	L	Y
B1AR	M	V	G	D	N	Y
B2AR	M	V	G	D	N	Y
D2R	L	M	V	D	T	Y
M2R	A	M	Y	D	Y	Y
M4R	T	M	Y	D	Y	Y
NTS1R	Y	M	E	R	N	Y
CB1	S	F	S	V	S	L
CB2	C	F	S	V	S	L

Appendix Figure S6. Sequence conservation of residues for hydrophobic packing in C3aR and class A GPCRs. Sequence alignment of human C3aR with 29 related human class A GPCRs is shown. Conservation at positions involved in hydrophobic packing of C3aR is indicated. BW number and the C3aR residues at each position are shown below the alignment (left). Position 7.43, which is mainly conserved as Y or F, is highlighted in red (right). Sequences of human C3aR and other 29 GPCRs were aligned using MAFFT with L-INS-i strategy (Katoh & Stadnley, 2013).

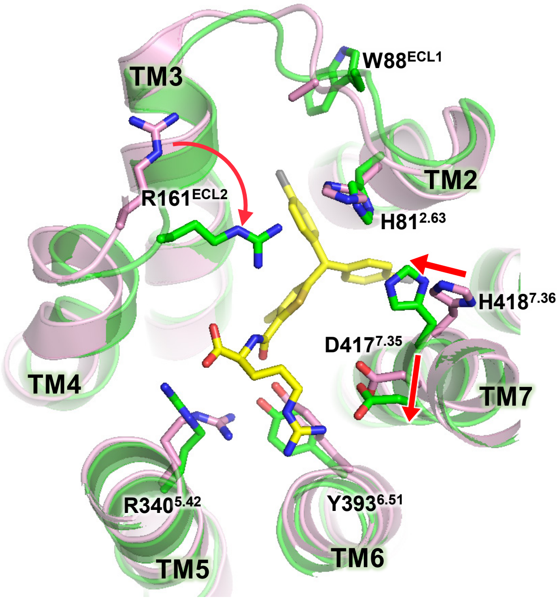

Appendix Figure S7. Structural comparison of apo-C3aR-G$_o$ and JR14a-bound C3aR-G$_i$. The active state apo-C3aR structure (PDB: 8I9S) was aligned with the JR14a-bound C3aR active structure. The active state apo-C3aR (pink) and JR14a-bound C3aR (green) structures are superimposed, with JR14a shown in yellow. Side chains of residues interacting with JR14a and their corresponding residues in the apo structure are displayed as sticks. Red arrows indicate conformational differences in the binding pocket residues between the two structures.

A

Ligand	BRET assay		cAMP assay	
	pEC_{50} ± SEM	N	pIC_{50} ± SEM	N
C3a	8.51 ± 0.08	4	8.56 ± 0.04	21
JR14a	8.34 ± 0.34	4	8.34 ± 0.06	14
EP54			8.27 ± 0.07	4
C3a 63-77			7.31 ± 0.12	4

B

C3aR Mutants	JR14a				Surface Expression (%)
	pIC_{50} ± SEM	N	ΔpIC_{50} ± SE of Diff.	Adjusted p-value	
WT	8.34 ± 0.06	14			100 ± 0
$S26^{1.35}A$	8.42 ± 0.19	3	0.14 ± 0.07	ns	98.2 ± 3.5
$L30^{1.39}A$	8.27 ± 0.17	3	-0.01 ± 0.01	ns	82.2 ± 3.3
$H81^{2.63}A$	7.91 ± 0.10	4	-0.54 ± 0.05	*	100.5 ± 5.5
$L82^{2.64}A$	8.69 ± 0.11	3	0.40 ± 0.14	ns	80.7 ± 1.7
$I98^{3.28}A$	8.05 ± 0.08	3	-0.36 ± 0.02	*	87.2 ± 9.2
$P99^{3.29}A$	8.39 ± 0.09	3	0.10 ± 0.06	ns	81.2 ± 7.9
$I102^{3.32}A$	8.16 ± 0.09	3	-0.13 ± 0.07	ns	93.4 ± 4.8
$V103^{3.33}A$	8.08 ± 0.22	3	-0.20 ± 0.05	ns	100.0 ± 5.7
$M106^{3.36}A$	8.49 ± 0.22	3	0.21 ± 0.09	ns	84.8 ± 2.9
$R161^{ECL2}A$	8.64 ± 0.10	3	0.09 ± 0.04	ns	113 ± 24
$Y174^{ECL2}A$	8.77 ± 0.18	3	0.22 ± 0.09	ns	90 ± 13
$R340^{5.42}A$	6.27 ± 0.32	3	-2.28 ± 0.14	*	104 ± 17
$Y393^{6.51}A$	6.50 ± 0.05	3	-2.05 ± 0.13	*	90.6 ± 9.0
$D417^{7.35}A$	6.37 ± 0.07	3	-2.18 ± 0.05	**	96.2 ± 4.1
$H418^{7.36}A$	8.60 ± 0.16	4	0.14 ± 0.08	ns	88.1 ± 4.8
$I421^{7.39}A$	6.66 ± 0.10	4	-1.80 ± 0.06	***	83.1±1.9
$I102^{3.32}A/I421^{7.39}A$	5.77 ± 0.09	3	-2.34 ± 0.08	**	98.0 ± 3.3

C3aR Mutants	C3a				Surface Expression (%)
	pIC_{50} ± SEM	N	ΔpIC_{50} ± SE of Diff.	Adjusted p-value	
WT	8.56 ± 0.04	21			100 ± 0
$S26^{1.35}A$	8.68 ± 0.13	4	-0.018 ± 0.007	ns	98.2 ± 3.5
$L30^{1.39}A$	8.57 ± 0.05	4	0.019 ± 0.009	ns	82.2 ± 3.3
$H81^{2.63}A$	8.11 ± 0.06	7	-0.39 ± 0.05	**	100.5 ± 5.5
$L82^{2.64}A$	8.71 ± 0.10	4	0.17 ± 0.05	ns	80.7 ± 1.7
$I98^{3.28}A$	8.38 ± 0.05	7	-0.11 ± 0.02	*	87.2 ± 9.2
$P99^{3.29}A$	8.18 ± 0.07	4	-0.36 ± 0.06	*	81.2 ± 7.9
$I102^{3.32}A$	8.47 ± 0.11	4	-0.08 ± 0.06	ns	93.4 ± 4.8
$V103^{3.33}A$	8.46 ± 0.11	4	-0.09 ± 0.05	ns	100.0 ± 5.7
$M106^{3.36}A$	8.51 ± 0.08	4	-0.03 ± 0.03	ns	84.8 ± 2.9
$R161^{ECL2}A$	Not calculated	3			113 ± 24
$Y174^{ECL2}A$	8.04 ± 0.18	5	-0.59 ± 0.09	*	90 ± 13
$R340^{5.42}A$	7.21 ± 0.15	6	-1.39 ± 0.06	****	104 ± 17
$Y393^{6.51}A$	7.26 ± 0.21	6	-1.34 ± 0.2	**	90.6 ± 9.0
$D417^{7.35}A$	Not calculated	3			96.2 ± 4.1
$H418^{7.36}A$	8.49 ± 0.04	7	-0.008 ± 0.011	ns	88.1 ± 4.8
$I421^{7.39}A$	8.61 ± 0.07	7	0.12 ± 0.03	ns	83.1±1.9
$I102^{3.32}A/I421^{7.39}A$	7.99 ± 0.05	3	-0.51 ± 0.05	*	98.0 ± 3.3

Appendix Table S1. Ligand-induced G_i recruitment and cAMP signaling of C3aR. A. The potency (EC_{50} or IC_{50}) of ligands in G_i recruitment to C3aR and in cAMP signaling is indicated with standard error of the mean (S.E.M). **B.** A table summarizes pIC_{50} values for cAMP signaling of C3aR mutants. For each C3aR mutant, pIC_{50} with S.E.M., number of independent experiments, ΔpIC_{50} with standard error of difference, and relative surface expression with S.E.M. are provided. Statistical significance is indicated by p-value ranges. (ns P>0.05, * P≤0.05, ** P≤0.01, *** P≤0.001, **** P≤0.0001)

	EMD-60785, PDB 9IPY<br>JR14a-bound human C3aR	EMD-60836, PDB 9ISI<br>apo human C3aR	EMD-60782<br>EMD-60783 (Gi1 focused map)<br>EMD-60784 (JR14a-C3aR focused map),<br>PDB 9IPV<br>JR14a-C3aR-Gαiβγ–scFv16
**Data collection and processing**			
Magnification (nominal)	105,000	105,000	96,000
Microscopy	Titan Krios 300 kV	Titan Krios 300 kV	Titan Krios 300 kV
Detector	Gatan K3 BioQuantum, 20eV slit	Gatan K3 BioQuantum, 20eV slit	TFS Falcon4i
Electron exposure (e/Å^2)	60.8	62.6	60
Number of frames per movie	50	54	50
Defocus range	- 0.9, -1.0, -1.1,-1.2,-1.3,-1.5,-1.7 µm	- 0.9, -1.0, -1.1, -1.3,-1.5,-1.7 µm	-1.0, -1.1, -1.3,-1.5,-1.7,-1.9 µm
Pixel size	0.85 Å/pix	0.848 Å/pix	0.81 Å/pix
Symmetry imposed	C1	C1	C1
Initial particle images picked	1.4 M particles out of 8,699 movies	1.5 M particles out of 15,338 movies	0.5 M particles out of 7,008 movies
Final particle images for reconstructio n	342,058	349,007	372,365
Map resolution (Å)	3.5 Å	3.6 Å	2.5 Å
Map sharpening	-180.9	-113.9	-83.6
**Model refinement**			
Initial model used	AlphaFold2 (C3aR)<br>(Jumper *et al*, 2021)	AlphaFold2 (C3aR)<br>(Jumper *et al*, 2021)	AlphaFold2 (C3aR)<br>(Jumper *et al*, 2021),<br>7VGX (heterotrimer G protein)
**Model composition**			
Non-hydrogen atoms	2100	1955	8822
Protein residues	264	252	1124
Ligands	JR14a : 1	0	JR14a : 1
**Protein geometry**			
All-atom contacts (Clash score)	5.40	9.20	9.57
Poor rotamers [%]	0	0	0
CaBLAM outliers [%]	2.08	2.16	1.84
Ramanchandra favored [%]	94.84	97.52	96.02
Ramanchandra allowed [%]	5.16	2.48	3.98
Ramanchandra outliers [%]	0	0	0
RMS deviation (bond lengths)	0.006	0.005	0.004
RMS deviation (bond angles)	1.052	0.999	0.648
**Model validation**			
Model-map FSC at 0.5 correlation [Å]	3.7	3.99	2.7
MolProbity score (Chen *et al*, 2010)	1.65	1.59	1.78
EMRinger score (Barad *et al*, 2015)	1.15	1.52	3.35

Appendix Table S2. Cryo-EM data collection, refinement and validation statistics.

References

Barad BA, Echols N, Wang RY-R, Cheng Y, DiMaio F, Adams PD, Fraser JS (2015) EMRinger: side chain–directed model and map validation for 3D cryo-electron microscopy. *Nature Methods* 12: 943-946

Chen VB, Arendall WB, 3rd, Headd JJ, Keedy DA, Immormino RM, Kapral GJ, Murray LW, Richardson JS, Richardson DC (2010) MolProbity: all-atom structure validation for macromolecular crystallography. *Acta Crystallogr D Biol Crystallogr* 66: 12-21

Durrant JD, de Oliveira CAF, McCammon JA (2011) POVME: An algorithm for measuring binding-pocket volumes. *Journal of Molecular Graphics and Modelling* 29: 773-776

Durrant JD, Votapka L, Sørensen J, Amaro RE (2014) POVME 2.0: An Enhanced Tool for Determining Pocket Shape and Volume Characteristics. *Journal of Chemical Theory and Computation* 10: 5047-5056

Jumper J, Evans R, Pritzel A, Green T, Figurnov M, Ronneberger O, Tunyasuvunakool K, Bates R, Zidek A, Potapenko A *et al* (2021) Highly accurate protein structure prediction with AlphaFold. *Nature* 596: 583-589

Katoh K, Standley DM (2013) MAFFT Multiple Sequence Alignment Software Version 7: Improvements in Performance and Usability. *Molecular Biology and Evolution* 30: 772-780
