# Supplementary figures and images for: Structural insights into small-molecule agonist recognition and activation of complement receptor C3aR
Source: EMBO J. 2025 Apr 7;44(10):2803–26. doi: 10.1038/s44318-025-00429-w (PMC12084609; doi:10.1038/s44318-025-00429-w)

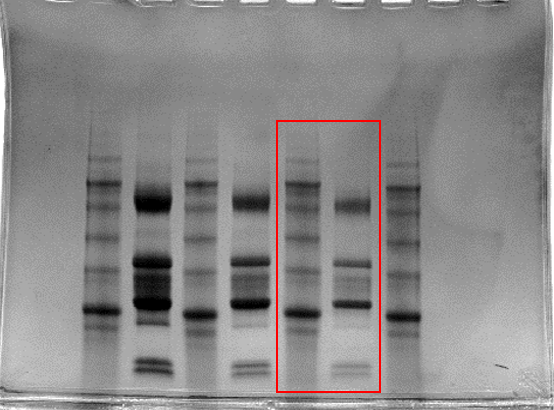

Supplement: Supplementary file 4 — Figure Source Data for EV and Appendix Figures [file 44318_2025_429_MOESM4_ESM.zip › Figure EV2/2A/apo-C3aR-BRIL complex SDS-PAGE.tif]

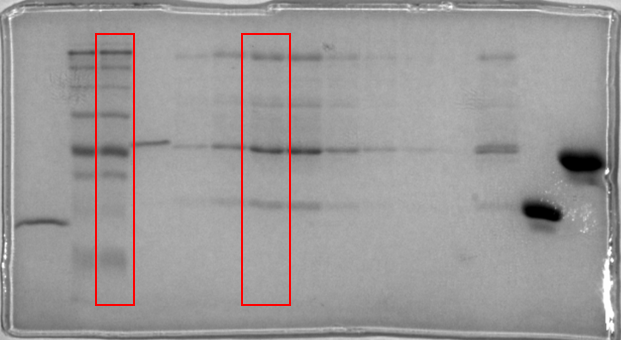

Supplement: Supplementary file 4 — Figure Source Data for EV and Appendix Figures [file 44318_2025_429_MOESM4_ESM.zip › Figure EV3/3A/JR14a-C3aR-BRIL complex SDS-PAGE.tif]

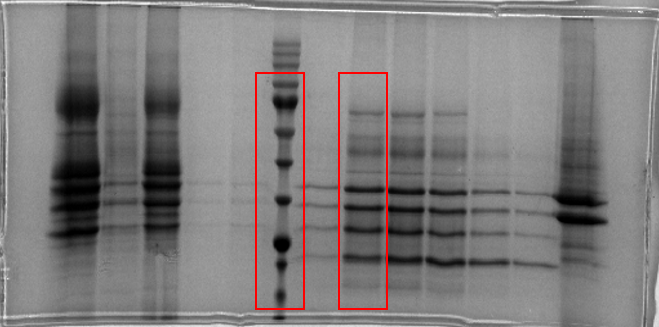

Supplement: Supplementary file 4 — Figure Source Data for EV and Appendix Figures [file 44318_2025_429_MOESM4_ESM.zip › Figure EV4/4A/JR14a-C3aR-Gi1 complex SDS-PAGE.tif]
