# Supplementary material for: Structural insights into small-molecule agonist recognition and activation of complement receptor C3aR
Source: EMBO J. 2025 Apr 7;44(10):2803–26. doi: 10.1038/s44318-025-00429-w (PMC12084609; doi:10.1038/s44318-025-00429-w)
Supplement: Supplementary file 5 — Expanded View Figures [file 44318_2025_429_MOESM5_ESM.pdf]

Expanded View Figures

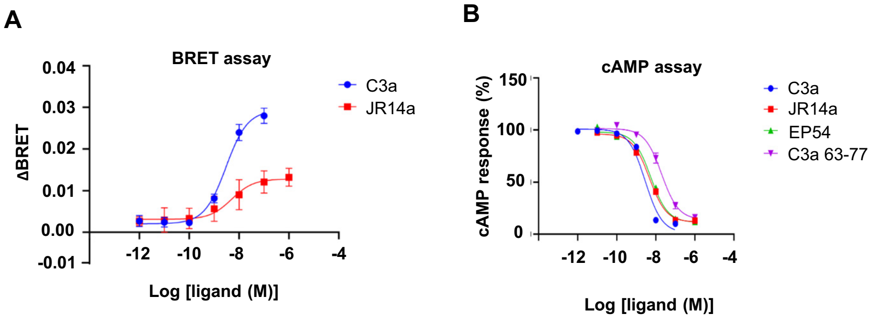

Figure EV1. Dose-response curves of C3aR-mediated G_i signaling in response to various ligands.

(**A**) G_i recruitment BRET assay was performed for C3a (blue circle) and JR14a (red rectangle). (**B**) cAMP response was assessed for C3a (blue circle), JR14a (red rectangle), EP54 (green triangle), and C3a 63–77 (purple inverted triangle). Each data point represents the mean ± standard error of the mean (S.E.M.) from n = 4–15 independent experiments, with detailed n values provided in Appendix Table S1. Source data are available online for this figure.

 © The Author(s)

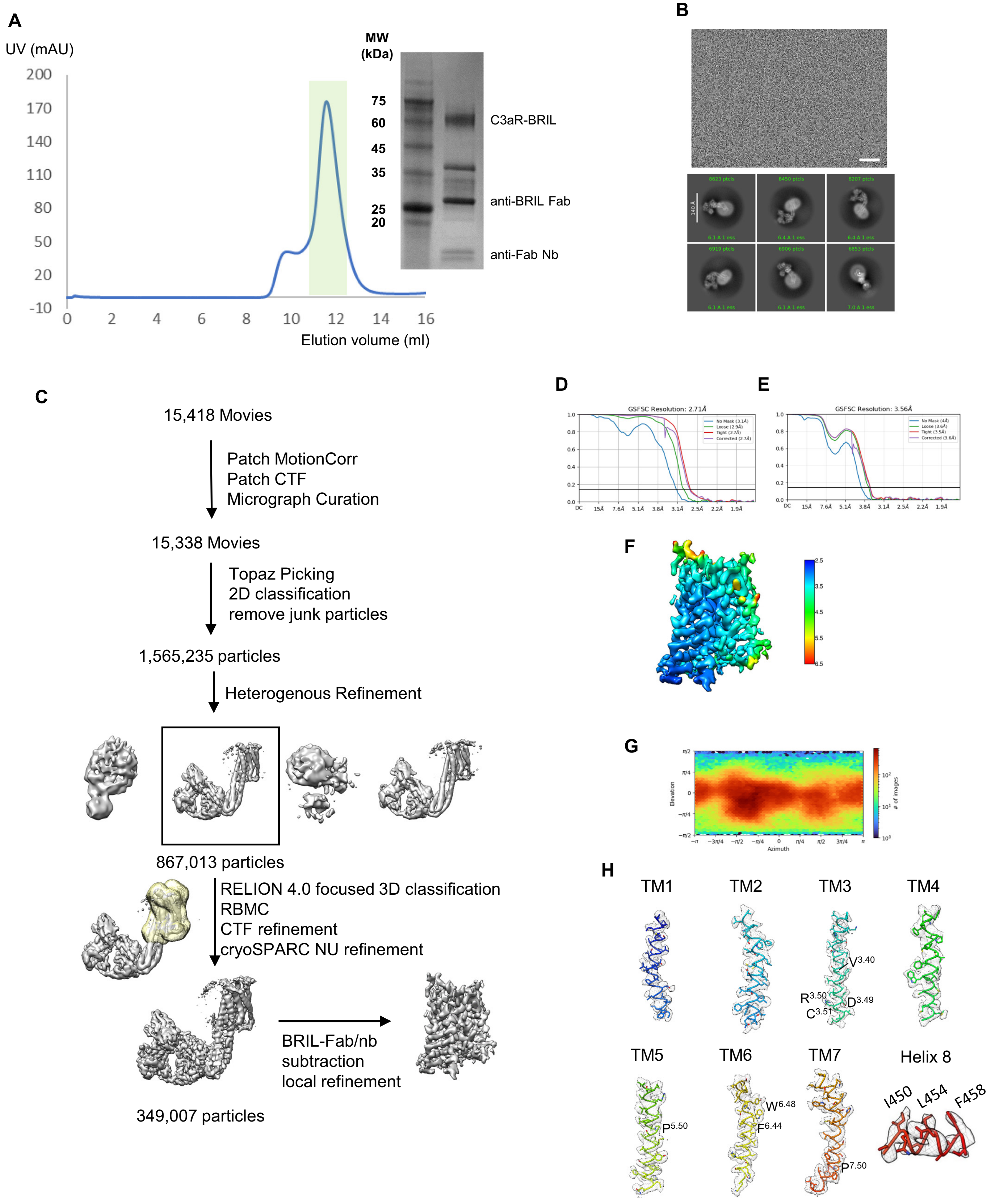
A
UV (mAU)
200
170
140
110
80
50
20
-10
0 2 4 6 8 10 12 14 16
Elution volume (ml)
MW (kDa)
75
60
45
35
25
20
C3aR-BRIL
anti-BRIL Fab
anti-Fab Nb
B
C
15,418 Movies
Patch MotionCorr
Patch CTF
Micrograph Curation
15,338 Movies
Topaz Picking
2D classification
remove junk particles
1,565,235 particles
Heterogenous Refinement
867,013 particles
RELION 4.0 focused 3D classification
RBMC
CTF refinement
cryoSPARC NU refinement
BRIL-Fab/nb subtraction
local refinement
349,007 particles
D
GSFSC Resolution: 2.71Å
E
GSFSC Resolution: 3.56Å
F
G
Elevation
Azimuth
H
TM1
TM2
TM3
TM4
V3.40
R3.50
D3.49
C3.51
TM5
TM6
TM7
Helix 8
P5.50
W6.48
F6.44
P7.50
I450
L454
F458

Figure EV2. Cryo-EM data analysis of apo C3aR – BRIL with anti-BRIL Fab and anti-Fab Nanobody complex.

(**A**) SEC profile (left) and SDS- PAGE (right) of purified apo C3aR–BRIL in complex with anti-BRIL Fab and anti-Fab Nb. (**B**) Representative micrograph (scale bar, 50 nm) and 2D average classes (scale bar, 140 Å). (**C**) Flowchart of data processing using cryoSPARC v4.5.1 and RELION 4.0. (**D**) FSC curves or cryo-EM maps for non-uniform refinement and (**E**) C3aR-focused local refinement. (**F**) Cryo-EM maps colored by local resolution of C3aR-focused local refinement. (**G**) The Euler angle distribution of final reconstructed local refinement map. (**H**) Density representation of TMs and helix8 are shown. Source data are available online for this figure.

© The Author(s)

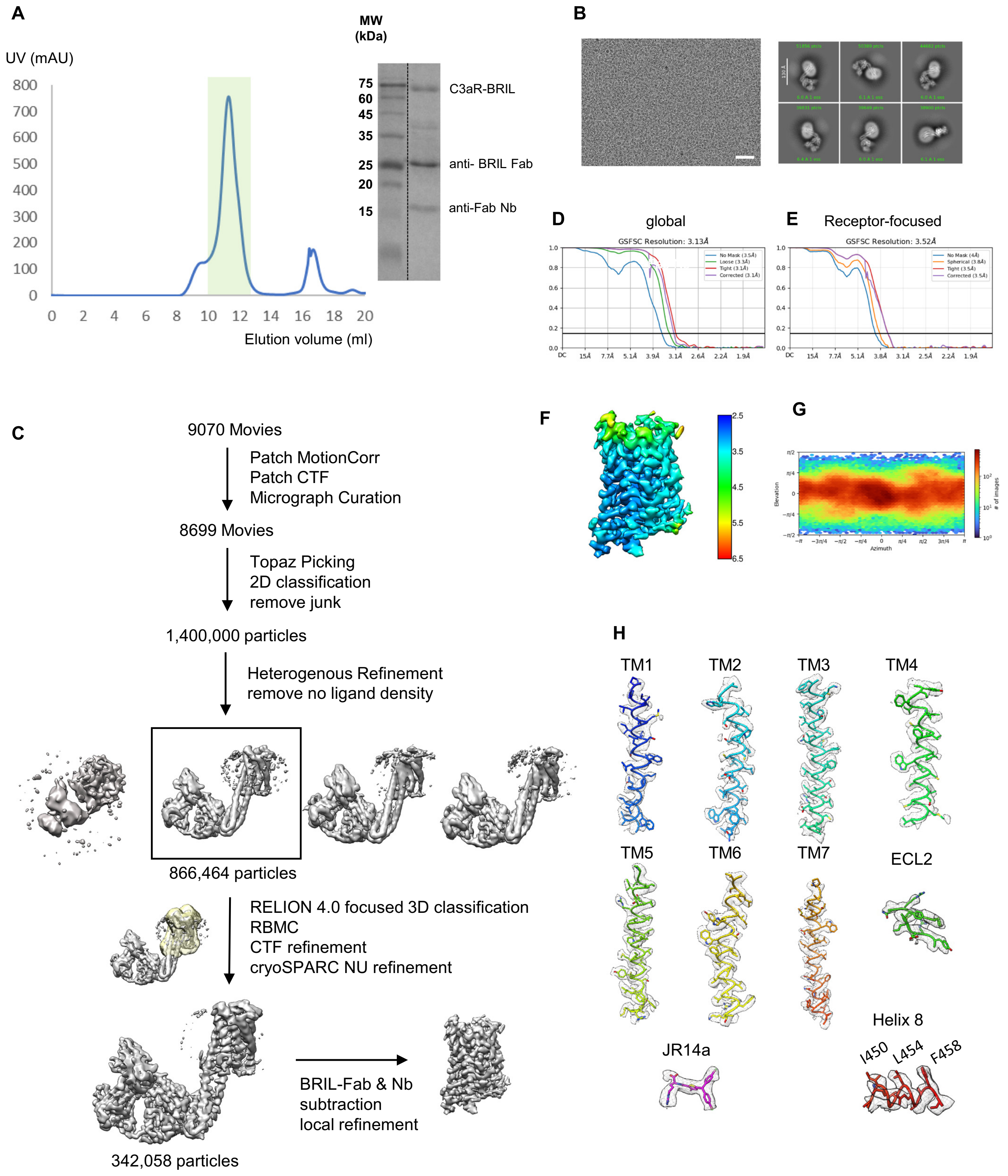
A
UV (mAU)
800
700
600
500
400
300
200
100
0
0 2 4 6 8 10 12 14 16 18 20
Elution volume (ml)
MW (kDa)
75
60
45
35
25
20
15
C3aR-BRIL
anti- BRIL Fab
anti-Fab Nb
B
D
global
GSFSC Resolution: 3.13Å
No Mask (3.5Å)
Loose (3.3Å)
Tight (3.1Å)
Corrected (3.1Å)
E
Receptor-focused
GSFSC Resolution: 3.52Å
No Mask (4Å)
Spherical (3.8Å)
Tight (3.5Å)
Corrected (3.5Å)
C
9070 Movies
Patch MotionCorr
Patch CTF
Micrograph Curation
8699 Movies
Topaz Picking
2D classification
remove junk
1,400,000 particles
Heterogenous Refinement
remove no ligand density
866,464 particles
RELION 4.0 focused 3D classification
RBMC
CTF refinement
cryoSPARC NU refinement
BRIL-Fab & Nb
subtraction
local refinement
342,058 particles
F
2.5
3.5
4.5
5.5
6.5
G
Elevation
Azimuth
# of images
H
TM1
TM2
TM3
TM4
TM5
TM6
TM7
ECL2
Helix 8
JR14a
I450
L454
F458

© The Author(s)

Figure EV3. Cryo-EM data analysis of JR14a-bound C3aR-BRIL fusion with anti-BRIL Fab and anti-Fab Nanobody complex.

(**A**) SEC profile (left) and SDS- PAGE (right) of purified JR14a-bound C3aR−BRIL in complex with anti-BRIL Fab and anti-Fab Nb. (**B**) Representative micrograph (scale bar, 50 nm) and 2D average classes (scale bar, 130 Å). (**C**) Flowchart of data processing using cryoSPARC v4.5.1 and RELION 4.0. (**D**) FSC curves for cryo-EM maps for non-uniform refinement and (**E**) C3aR-focused local refinement. (**F**) Cryo-EM maps colored by local resolution of C3aR-focused local refinement. (**G**) The Euler angle distribution of final reconstructed local refinement map. (**H**) Density representation of TMs, ECL2, JR14a and helix8 are shown. Source data are available online for this figure.

 © The Author(s)

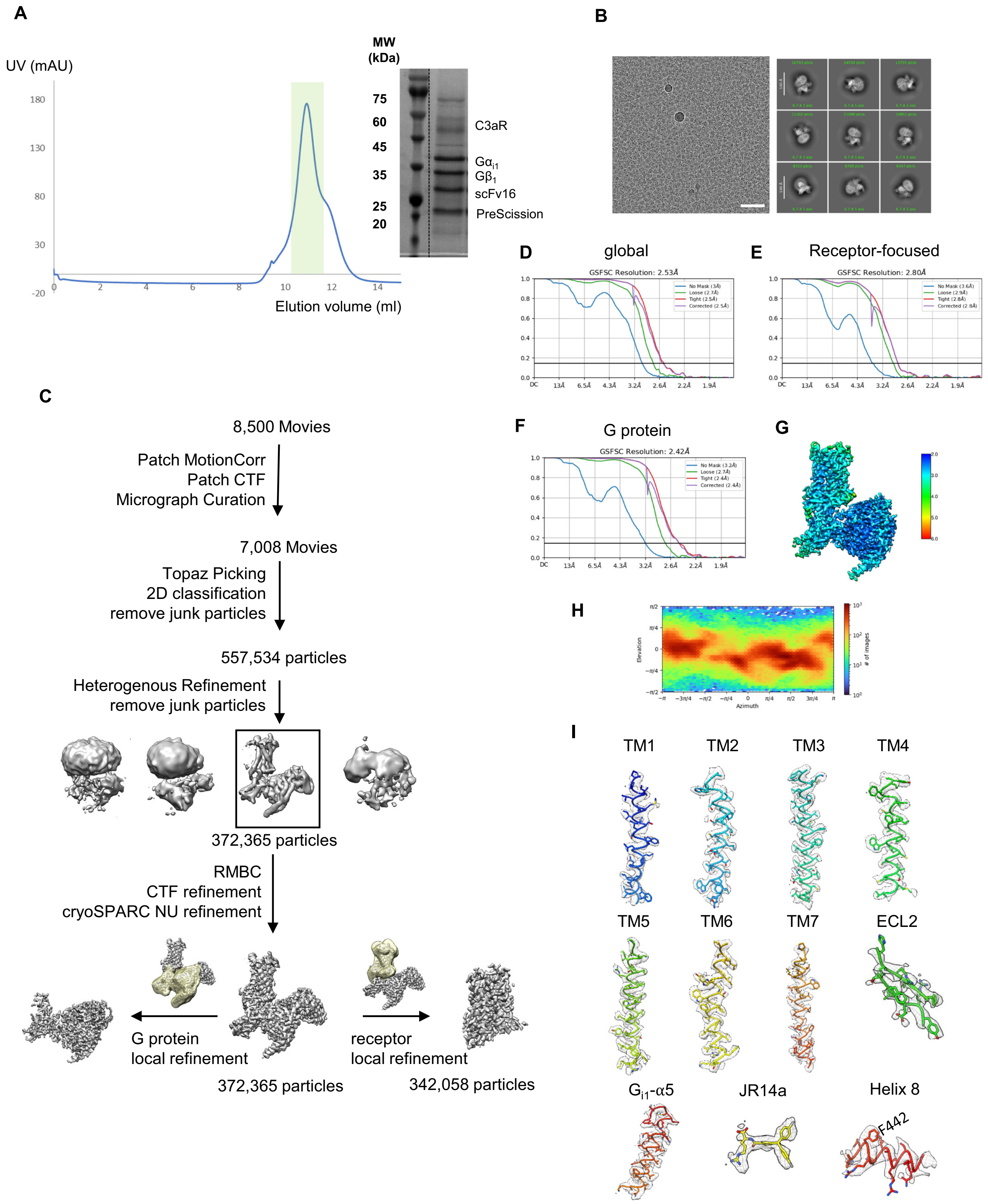
A
UV (mAU)
180
130
80
30
-20
0
2
4
6
8
10
12
14
Elution volume (ml)
MW (kDa)
75
60
45
35
25
20
C3aR
Gαi1
Gβ1
scFv16
PreScission
B
C
8,500 Movies
Patch MotionCorr
Patch CTF
Micrograph Curation
7,008 Movies
Topaz Picking
2D classification
remove junk particles
557,534 particles
Heterogenous Refinement
remove junk particles
372,365 particles
RMBC
CTF refinement
cryoSPARC NU refinement
G protein
local refinement
372,365 particles
receptor
local refinement
342,058 particles
D
global
GSFSC Resolution: 2.53Å
E
Receptor-focused
GSFSC Resolution: 2.80Å
F
G protein
GSFSC Resolution: 2.42Å
G
H
Elevation
Azimuth
# of images
I
TM1
TM2
TM3
TM4
TM5
TM6
TM7
ECL2
Gi1-α5
JR14a
Helix 8
F442

Figure EV4. Cryo-EM data analysis of JR14a-bound C3aR – G_i–scFv16 complex.

(**A**) SEC profile (left) and SDS- PAGE (right) of purified JR14a-bound C3aR – G_i–scFv16 complex. (**B**) Representative micrograph (scale bar, 50 nm) and 2D average classes (scale bar, 140 Å). (**C**) Flowchart of data processing using cryoSPARC v4.5.1. (**D**) FSC curves for cryo-EM maps for non-uniform refinement and (**E**) C3aR-focused local refinement and (**F**) G_i-focused local refinement. (**G**) Cryo-EM maps colored by local resolution of C3aR-focused local refinement. (**H**) The Euler angle distribution of final reconstructed local refinement map. (**I**) Density representation of TMs, ECL2, α5 helix of G_{i1}, JR14a and helix8 are shown. Source data are available online for this figure.

 © The Author(s)

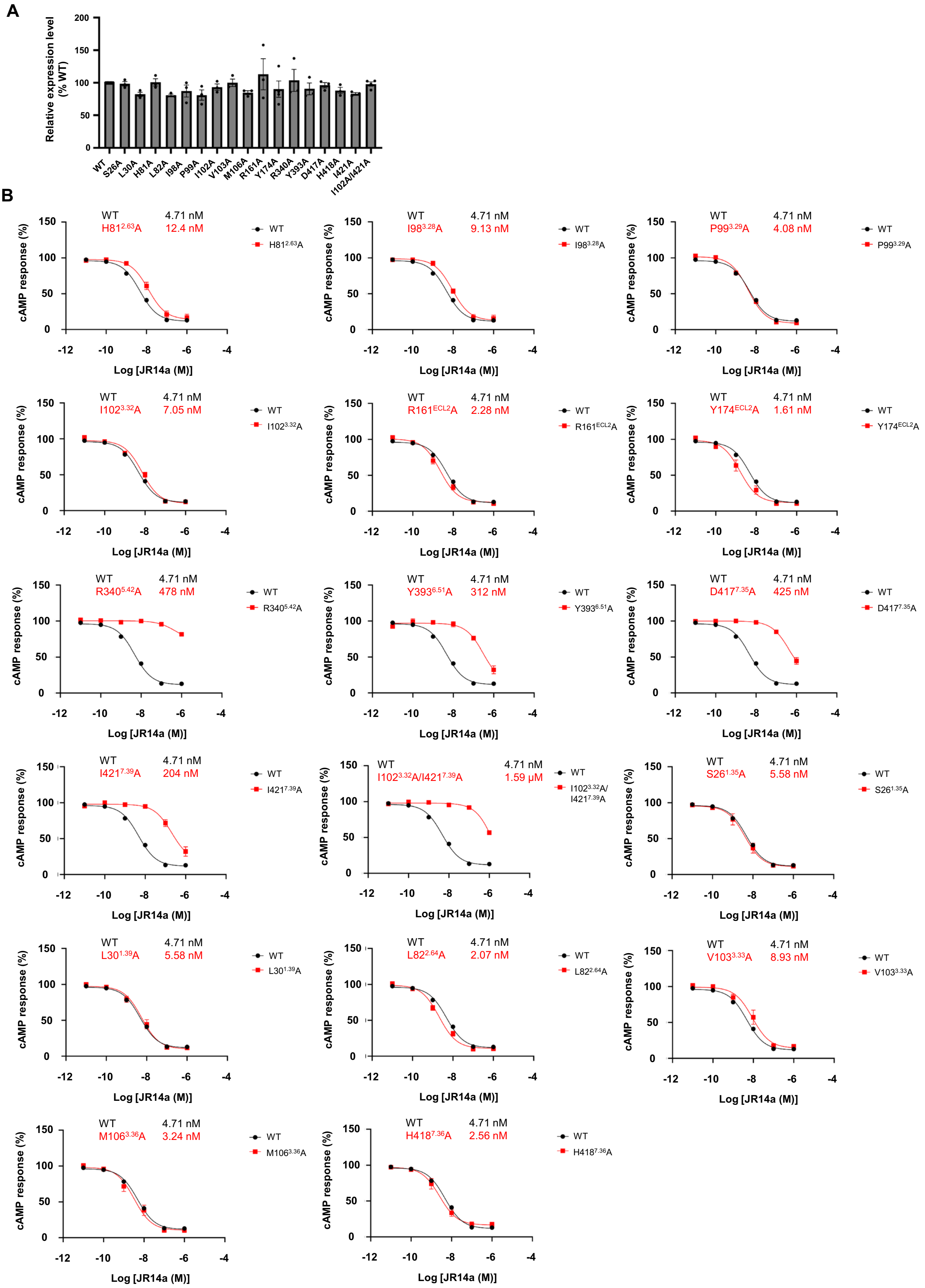
A
Relative expression level (% WT)
200
150
100
50
0
WT S26A L30A H81A L82A I98A P99A I102A V103A M106A R161A Y174A R340A Y393A D417A H418A I421A I102A/I421A
B
WT 4.71 nM
H81$^{2.63}$A 12.4 nM
I98$^{3.28}$A 9.13 nM
P99$^{3.29}$A 4.08 nM
I102$^{3.32}$A 7.05 nM
R161^{ECL2}A 2.28 nM
Y174^{ECL2}A 1.61 nM
R340$^{5.42}$A 478 nM
Y393$^{6.51}$A 312 nM
D417$^{7.35}$A 425 nM
I421$^{7.39}$A 204 nM
I102$^{3.32}$A/I421$^{7.39}$A 1.59 μM
S26$^{1.35}$A 5.58 nM
L30$^{1.39}$A 5.58 nM
L82$^{2.64}$A 2.07 nM
V103$^{3.33}$A 8.93 nM
M106$^{3.36}$A 3.24 nM
H418$^{7.36}$A 2.56 nM
cAMP response (%)
150
100
50
0
-12 -10 -8 -6 -4
Log [JR14a (M)]

Figure EV5. Quantification of surface expression and G_i signaling of C3aR mutants.

(**A**) Surface ELISA of C3aR WT and mutants. For each data, bars and error bars indicate the means and the standard errors of the mean (S.E.M.) of 3–4 independent experiments, respectively. (**B**) cAMP response of C3aR mutants. For each C3aR mutant, relative cAMP response (%) and IC_{50} was calculated and compared with WT using GraphPad Prism 10.1.2. For each data, points and error bars indicate the means and the standard errors of the mean (S.E.M.) of 3–4 independent experiments, respectively. Source data are available online for this figure.

 © The Author(s)
